# IGFBP-1 and IGF-I in relation to adiposity and mortality from midlife to old age in the Swedish Adoption/Twin Study of Aging

**DOI:** 10.1038/s41366-025-01773-x

**Published:** 2025-04-05

**Authors:** Moira S. Lewitt, Ida K. Karlsson, Nancy L. Pedersen

**Affiliations:** 1 School of Health and Life Sciences, University of the West of Scotland, Paisley, PA1 2BE UK; 2 Department of Medical Epidemiology and Biostatistics, Karolinska Institutet, 171 77 Solna, Sweden

**Keywords:** Obesity, Risk factors

## Abstract

**Background/Objectives:**

Insulin-like growth factor-binding protein (IGFBP)-1 is a marker of insulin resistance. Lower IGFBP-1 is associated with increased adiposity. The aims of this study were to determine whether IGFBP-1 and its ligand, IGF-I, are associated with weight and waist measurements across mid-life to old age, and predict survival.

**Subjects/Methods:**

The Swedish Adoption/Twin Study of Aging (SATSA) includes extensive in-person testing of same-sex twins over a 30-year period. The dataset of twins for which baseline fasting IGFBP-1 (*n* = 512; 251 twin pairs) and IGF-I (*n* = 537; 262 twin pairs) measurements were available (from 1986) was stratified by birth cohort. Latent growth curve modeling was used to determine whether BMI and waist-to-height ratio (WHtR) and their change, differed as a function of IGFBP-1 or IGF-I. Survival data was collected by linkage to the Swedish Tax Agency.

**Results:**

IGFBP-1 correlated inversely with insulin concentrations. There was a curvilinear relationship between BMI and age, increasing until 70–75 years and then declining, fitting a quadratic model. Lower IGFBP-1 was associated with higher BMI at the intercept, 73 years (1.8 kg/m^2^ per unit decrease in ln-IGFBP-1; *p* < 0.001). WHtR continued to increase beyond 70–75 years. Lower IGFBP-1 was associated with higher WHtR (3 cm/m per unit decrease in ln-IGFBP-1 at 73 years; *p* < 0.001). Associations weakened, but remained, after adjustment for ln-insulin. IGFBP-1 was not associated with the slope or shape of the trajectories. Between-within models, examining the associations within twin pairs, indicated these associations are explained in part by familial factors. There was no relationship between IGF-I and BMI or WHtR, or their trajectories. Neither IGFBP-1 nor IGF-I concentration predicted survival.

**Conclusion:**

Lower circulating IGFBP-1 concentrations are associated with increased adiposity but not change in adiposity, across the lifespan from middle to old age.

## Introduction

Insulin-like growth factor-binding protein (IGFBP)-1 is a member of a family of six proteins that bind IGFs and determine their availability to tissues and cells, and therefore the role of IGFs in growth and metabolism [1]. Hepatic synthesis of IGFBP-1 is transcriptionally inhibited by insulin. In obesity, low circulating IGFBP-1 concentrations are associated with increased portal insulin levels and increased IGF bioavailability [2, 3]. Multiple other factors regulate IGFBP-1; for example, IL-1β and TNFα are stimulatory, so that IGFBP-1 is implicated in chronic inflammatory states and during critical illness. IGFBP-1 also has IGF-independent actions, through its binding an Arg-Gly-Asp (RGD) integrin binding motif, that may improve insulin sensitivity [4].

The relative importance of genetic and environmental influences on IGFBP-1 and IGF-I concentrations has previously been evaluated in middle aged and older Swedish twins, reared apart and reared together. In the population-based Swedish Adoption/Twin Study of Aging (SATSA), heritability estimates were 36% for IGFBP-1 and 63% for IGF-I [5], and insulin explained 28% of the non-genetic variation in IGFBP-1 [6]. It is more than 30 years since these measurements were taken in the first wave of SATSA recruitment. The aim of this study was to determine the relationship between those baseline IGFBP-1 and IGF-I measurements with long-term measures of adiposity and survival. Specifically, we ask whether IGFBP-1 or IGF-I predict (1) trajectories of BMI and waist measurement from midlife to late-life and (2) survival.

## Methods

### Participants

The SATSA dataset was drawn from the Swedish Twin Registry and includes same-sex twin pairs born in Sweden separated before the age of 11 years and reared apart. Control twin pairs were reared together and matched on sex, date of birth and county of birth. In 1986, twin pairs aged 50 and above were invited to interview and health examination, including a fasting blood sample [7]. Subsequently there were up to ten rounds of in-person testing at approximately 3-year intervals over a 30-year period. Zygosity was determined by questionnaire and serology, and in some cases genotyping. There were more dizygotic (DZ) than monozygotic (MZ) twins in the SATSA dataset (68%) [7]. The study was approved by the ethics committees of The Karolinska Institute and the Swedish National Data Inspection Authority. All participants gave informed consent. All methods were performed in accordance with the relevant guidelines and regulations.

Data presented in this paper are from twins that had IGFBP-1 and/or IGF-I measurements in fasting blood samples taken at the first in-person testing. IGFBP-1 had been determined in 512 individuals (251 pairs), all of whom had baseline BMI measurement and, of these, 346 (160 pairs) also had baseline WHtR measurements. IGF-I was available in 535 individuals (262 twin pairs) and, of these, 534 (262 pairs) had BMI measurements, and 351 (164 pairs) also had waist measurements at baseline. Twins contributing to this dataset were born between 1900 and 1944. Notable, population mortality rates declined substantially across their lifetimes [8]. Survival data through to December 31 2018 was collected by linkage to the Swedish Tax Agency.

### Assays

Serum concentrations of IGFBP-1 and IGF-I were determined by in-house radioimmunoassays according to previously described methods [9, 10], and values were previously reported [5, 6]. In brief, samples were extracted with acid ethanol before assay for IGF-I, and truncated (des1-3) IGF-I was used as the ligand. The intra- and inter-assay coefficients of variation were 3% and 10%, respectively, for IGFBP-1 and 4% and 11% for IGF-I. Insulin concentrations were measured using a commercial radioimmunoassay (RIA 100, Pharmacia, Uppsala, Sweden), and values were previously reported [5, 6].

### Anthropometric measures

Height, weight, and waist were measured by trained research nurses. BMI was calculated as weight (kg)/height(m)^2^. Waist was measured at the midpoint between the lower costal margin and the iliac crest, and was expressed as a ratio with height (WHtR = waist,cm/height,cm).

### Statistical analyses

To account for the decline in mortality rates across the age spectrum of the population [8], the sample was divided into three cohorts for analyses; those born 1900–1917 (age range 69–88 years), 1918–1925 (61–70 years) and 1926–1944 (42–62 years).

Statistical analyses were conducted in STATA 15.1 and SPSS Statistics 25. Statistical significance was set at *α* = 0.05. IGFBP-1, IGF-I and insulin had skewed distributions and were ln-transformed for statistical analysis. The relationship of baseline values between twins was determined with paired *t*-tests and Pearson correlations, and between cohorts with Pearson Chi Square or Analysis of Variance (and Tukey post hoc test), as appropriate. Results in the tables are presented as mean ± 95% confidence intervals (CI). Hierarchical multiple regression was used to determine which variables predicted a significant amount of the variance in ln-IGFBP-1, ln-insulin and ln-IGF-I concentrations.

Latent growth curve models [11] were fitted to the data to determine whether BMI and WHtR trajectories differed as a function of baseline IGFBP-1 or IGF-I. Linear mixed models with random effects for intercept, linear and quadratic age were tested, and the best fitting model selected based on Akaike information criterion. Mean-centered age (73 years) appeared optimum, and use of other centering ages did not change the models’ fit. This centering age also corresponds to the age of change in BMI from our previous work [12, 13]. Between-within models [14] were also fitted to the data. The between-pair estimate represents the average effect within the population while the within-pair effect represents the effect of IGFBP-1 or IGF-I that is independent of familial factors. Sex and birth cohort were added as fixed effects in all models. Relatedness between the twins was accounted for by including individual IDs nested within twin pair IDs as random effects on the intercepts and slopes.

The associations between IGFBP-1 or IGF-I concentrations at baseline, and survival were tested in Cox proportional hazards regression models, using the Breslow method for ties. Age was used as the underlying time scale, and individuals followed from baseline to age at death or end of follow-up, whichever occurred first. The models were thereby adjusted for age, and we further adjusted for sex and birth cohort by including them as covariates. Relatedness between the twins was accounted for with robust standard errors. Inspection of survival curves did not indicate time-varying effects, and we modeled average hazard rate ratios over time.

## Results

### Baseline data

Baseline data is presented in Table 1. The overabundance of dizygotic twins in the 1918–1925 and 1926–1944 cohorts, compared to the 1900–1917 cohort was similar in magnitude to that in the wider SATSA dataset [7]. In the 1918–1925 cohort there were fewer females compared to the other groups and this is likely to account for the lower height in that group. There were no differences in BMI or WHtR between cohorts.

**Table 1:** Baseline data and age at death by birth cohort.

	Total sample	1900–1917	Cohort 1918–1925	1926–1944	*F* (df 2), *p* between cohorts
Number (twin pairs)	535 (262)	178 [87]	207 (102)	150 [73]	8.784, 012*
Monozygotic (%)	39%	47%	38%	37%	7.086, 029*
Female (%)	60%	73%	43%	65%	25.030, 000*
Reared apart (%)	46%	43%	42%	52%	4.032, 133*
Age, yr	65.8 65.1–66.5, 535	74.7^a^ 74.0–75.3, 178	65.5^b^ 64.1–65.8, 207	55.8^c^ 55.0–56.5, 150	975.508, 000^†^
Age at death, yr	84.8 84.1–85.6, 436	87.6^a^ 86.3–88.8, 175	84.5^b^ 82.9–86.1, 190	78.2^c^ 78.6–81.3, 71	42.764, 000^†^
Height, cm	166.2 165.3–167.0, 534	161.9^a^ 160.5–163.3, 177	168.1^b^ 166.8–169.3, 207	168.6^b^ 167.0–170.1, 150	27.138, 000^†^
BMI, kg/m^2^	25.5 25.2–25.9, 534	25.2 24.7–25.8, 177	25.9 25.3–26.4, 207	25.4 24.8–26.1, 150	1.511, 222^†^
WHtR	0.533 527–0.540, 531	0.539 527–0.551, 114	0.535 525–0.544, 149	0.524 512–0.535, 88	1.719, 181^†^
IGFBP-1, μg/L^‡^	35.8 33.7–38.1, 512	42.1^a^ 38.4–46.2, 174	33.3^b^ 31.0–35.8, 199	34.4^b^ 30.4–36.8, 139	9.716, 000^†^
Insulin, pmol/L^‡^	83.1 78.3–88.2, 433	88.1^a^ 79.6–97.4, 138	82.4^a^ 76.1–89.2, 173	66.2^b^ 60.7–72.1, 122	9.906, 000^†^
IGF-I, μg/L^‡^	130.8 126.2–135.6, 535	119.3^a^ 113.0–125.8, 178	134.0^b^ 128.4–139.9, 207	151.0^c^ 143.0–159.5, 150	19.984, 000^†^

Mean, 95% CI, *n*; WHtR, waist-to-height ratio; ^‡^geometric mean of fasting values; *Pearson Chi Square; ^†^ANOVA; ^a,b,c^Tukey post hoc test (identical superscripts indicate no difference).

*BMI* weight(kg)/height(m)^2^, *WHtR* waist/height, *IGF* insulin-like growth factor, *IGFBP-1* IGF-binding protein.

**Fig. 1:**
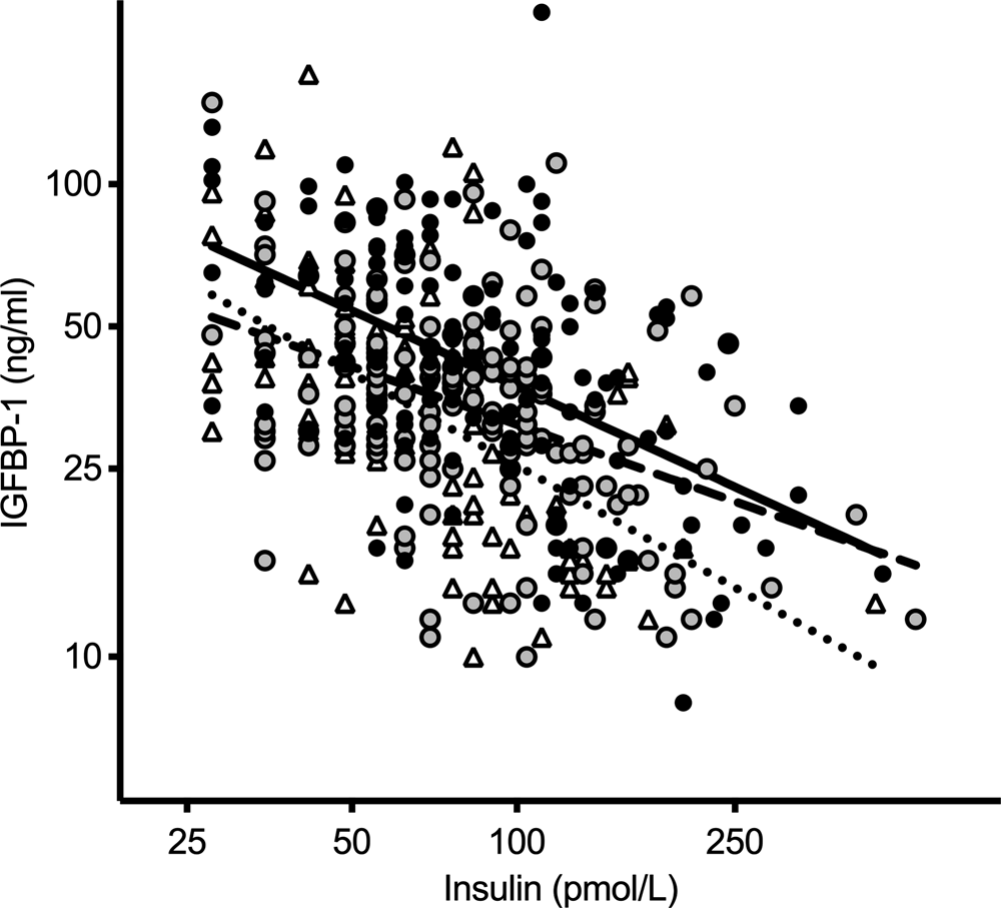
Relationship between fasting serum IGFBP-1 and insulin concentrations in the 1900–1917 cohort (closed circles and solid line, *n* = 135), 1918–1925 cohort (open circles and broken line, *n* = 165) and 1926–1944 cohort (triangles and dotted line, *n* = 113). Regression lines and correlation coefficients for the relationships (outlier excluded from analysis): 1900–1917 cohort: ln-IGFBP-1 = 4.911 − 0.478*ln-insulin (Pearson *r* = 0.484, *p* < 0.0001). 1918–1925 cohort: ln-IGFBP-1 = 4.527 − 0.410*ln-insulin (Pearson *r* = 0.421, *p* < 0.0001). 1926–1944 cohort: ln-IGFBP-1 = 4.953 − 0.642*ln-insulin (Pearson *r* = 0.547, *p* < 0.0001).

Fasting IGFBP-1 concentrations were highest in the 1900–1917 cohort. Unexpectedly, given the well-documented inverse relationship between insulin and IGFBP-1 [15–18], insulin concentrations were also highest in the 1900–1917 cohort. The inverse relationship between IGFBP-1 and insulin concentrations was preserved for each cohort (Fig. 1). There was no significant difference in the slopes of the regression lines shown in Fig. 1 (*F*(2406) = 2.127, *p* = 0.109). However, there was a significant difference in the intercepts (*F*(2408) = 13.54, *p* < 0.001), with an upward shift in the regression lines from the youngest 1926–1944 to the oldest 1900–1917 cohort. In hierarchical multiple regression, ln-insulin introduced at Step 1 explained 19% of the variance in lnIGFBP-1 (*F*(1415) = 94.024, *p* < 0.001). After entry of age and sex at Step 2, the total variance in ln-IGFBP-1 explained by the model increased to 26% (*F*(3413) = 48.522, *F* change *p* < 0.001). There was no significant change with the addition of cohort, rearing status and zygosity at Step 3 (*F*(6410) = 24.948, *F* change *p* = 0.282). In the final model, ln-insulin (*β* = −0.513, *p* < 0.001), age (*β* = 0.013, *p* = 0.039) and female sex (*β* = 0.138, *p* < 0.006) significantly predicted ln-IGFBP-1.

In hierarchical multiple regression, BMI introduced at Step 1 explained 22% of the variance in ln-insulin (*F*(1,430) = 120.975, *p* < 0.001). After entry of age and sex at Step 2, the total variance in ln-insulin explained by the model increased to 26% (*F*(3,428) = 50.953, *F* change *p* < 0.001). There was no significant change with the addition of cohort, rearing status and zygosity at Step 3 (*F*(6,425), *F* change *p* = 0.824). In the final model, only BMI (*β* = −0.065, *p* < 0.001) significantly predicted ln-insulin, and there was a trend for age (*β* = −0.010, *p* = 0.087).

With ln-IGF-I as the dependent variable in hierarchical multiple regression, age introduced at Step 1 explained 9% of the variance (*F*(1,431) = 42.584, *p* < 0.001). After entry of ln-insulin and sex at Step 2, the total variance in ln-IGF-I explained by the model increased to 14% (*F*(3,429) = 23.894, *F* change *p* < 0.001). There was no significant change with the addition of cohort, rearing status and zygosity at Step 3 (*F*(6,426), *F* change *p* = 0.154). In the final model age (*β* = −0.021, *p* < 0.001), ln-insulin (*β* = 0.132, *p* < 0.001) and male sex (*β* = 0.075, *p* = 0.016) each significantly predicted ln-IGF-I.

The twin with the higher IGFBP-1 concentration he was compared with their co-twin with the lower IGFBP-1, in a pair-wise fashion, thus controlling for age and sex (Table 2). Six twin pairs had identical IGFBP-1 concentrations and were excluded from the analysis. There were significant paired sample correlations for ln-IGFBP-1, ln-IGF-I, ln-insulin, BMI and WHtR. The twins with higher IGFBP-1 had lower ln-insulin concentrations compared to their co-twins with lower IGFBP-1, in all cohorts. The twins with higher IGFBP-1 had lower BMI and WHtR measurements in the 1918–1925 and 1926–1944 cohorts and lower IGF-I concentrations in the 1900–1917 and 1926–1944 cohorts, compared to their co-twins with lower IGFBP-1.

**Table 2:** Paired comparisons on baseline data: twins with higher IGFBP-1 are compared with their co-twins with lower IGFBP-1.

	1900–1917			1918–1925			1926–1944		
	Higher BP-1	Lower BP-1	*p* (paired *t*) *r, *p*, *n*	Higher BP-1	Lower BP-1	*p* (paired *t*) *r, *p*, *n*	Higher BP-1	Lower BP-1	*p* (paired *t*) *r, *p*, *n*
IGFBP-1, μg/L^‡^	54.8 48.4–62.1	31.3 27.9–35.1	0.000 610, 000, 83	43.4 39.8–47.3	26.0 23.5–28.6	0.000 694, 000, 96	45.2 39.9–51.2	24.7 21.8–28.1	0.000 622, 000, 65
IGF-I, μg/L^‡^	112.2 103.8–121.3	129.0 119.9–138.8	0.000 511, 000, 83	130.3 122.3–138.7	137.7 129.2–146.7	0.095 472, 000, 96	139.5 127.7–152.4	147.5 136.5–159.4	0.000 502, 000, 65
Insulin pmol/L^‡^	76.3 66.02–88.1	96.7 83.2–112.3	0.004 429, 004, 61	76.4 68.1–85.7	96.8 85.5–109.6	0.002 239, 045, 71	56.7 50.7–63.3	78.5 66.7–92.4	0.000 400, 005, 47
BMI, kg/m^2^	25.2 24.3–26.0	25.6 24.7–26.4	0.413 296, 007, 83	25.2 24.4–26.0	26.4 25.7–27.2	0.000 632, 000, 96	24.1 23.3–24.8	26.3 25.4–27.2	0.000 356, 004, 65
WHtR	0.541 521–0.561	0.533 515–0.550	0.422 442, 001, 49	0.524 510–0.537	0.542 528–0.556	0.020 368, 002, 68	0.500 483–0.516	0.541 527–0.556	0.000 368, 023, 38
Age at death, yr	88.2 86.7–89.6	88.3 86.9–89.7	0.864 254, 023, 80	83.8 82.2–85.3	83.1 81.5–84.8	0.574 087, 445, 79	82.2 79.5–84.9	78.9 74.8–83.0	0.113 295, 207, 20

Mean, 95% CI; WHtR, waist-to-height ratio; *Pearson’s correlation coefficient; ^‡^geometric mean of fasting values.

*BMI* weight(kg)/height(m)^2^, *WHtR* waist/height, *IGF* insulin-like growth factor, *IGFBP-1* IGF-binding protein-1.

### BMI and WHtR trajectories

**Fig. 2:**
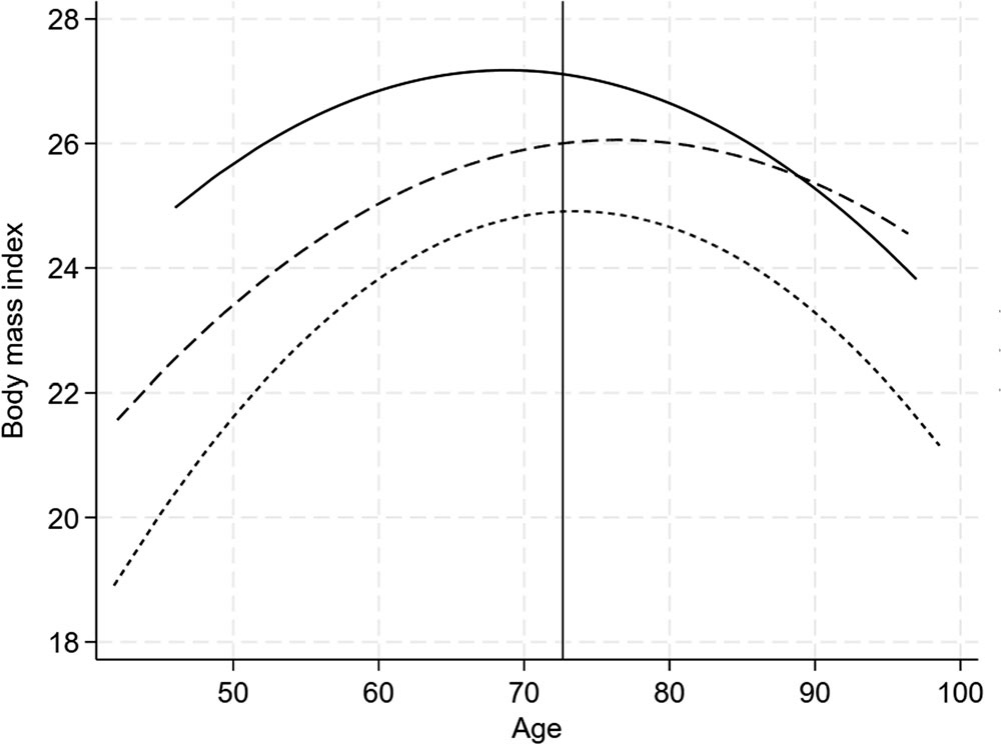
Longitudinal change in body mass index, stratified by tertiles of fasting IGFBP-1 concentration. The lowest (ln-IGFBP: 11.946–3.401), middle (ln-IGFBP: 3.434–3.784) and highest (ln-IGFBP: 3.807–5.142) tertile are represented by the solid, broken and dotted dashed line, respectively.

On average, each twin had 4.6 measures of BMI (range 1–10). The mid- to late-life trajectory of BMI is illustrated in Fig. 2, stratified by tertiles of fasting IGFBP-1 concentration. A quadratic model best fit the data. There was a curvilinear relationship between BMI and age, with BMI increasing until 70–75 years and then declining. When ln-IGFBP-1 was introduced into the model there was a significant inverse relationship with BMI at the intercept age of 73 years (Table 3) such that every unit increase in ln-IGFBP-1 (equivalent to 2.72 μg/L) was associated with 1.8 kg/m^2^ lower BMI. Adding adjustment for ln-insulin weakened, but did not abolish, the association. There was no association between ln-IGFBP-1 and the slope or shape of the BMI trajectory.

**Table 3:** Association between IGFBP-1 and IGF-I, and trajectories of BMI and WHtR.

	BMI				WHtR			
	Coeff	95% CI		*p*	Coeff	95% CI		*p*
IGFBP-1								
Intercept	**−1.809**	**−2.363**	**−1.245**	**0.000**	**−2.972**	**−3.884**	**−2.061**	**0.000**
Linear change	0.296	-0.044	0.635	0.088	0.406	-0.156	0.967	0.157
Quadratic change	-0.002	-0.004	0.001	0.149	-0.002	-0.006	0.002	0.279
Adjusted for insulin								
Intercept	**−1.450**	**−2.140**	**−0.759**	**0.000**	**−1.917**	**−3.060**	**−0.774**	**0.001**
Linear change	0.246	−0.109	0.602	0.175	0.465	−0.159	1.088	0.144
Quadratic change	0.002	−0.004	0.001	0.231	−0.003	−0.007	0.002	0.227
Between-within models								
Intercept								
Between-pair	**−2.343**	**−3.194**	**−1.492**	**0.000**	**−3.683**	**−5.031**	**−2.334**	**0.000**
Within-pair	**−1.497**	**−2.180**	**−0.814**	**0.000**	**−2.411**	**−3.574**	**−1.247**	**0.000**
Linear change								
Between-pair	−0.134	−0.433	0.166	0.381	−0.315	−0.304	0.934	0.319
Within-pair	**0.359**	**0.029**	**0.688**	**0.033**	**0.746**	**0.031**	**1.462**	**0.041**
Quadratic change								
Between-pair	0.001	−0.001	0.003	0.254	−0.001	−0.006	0.003	0.506
Within-pair	−0.002	−0.005	0.000	0.061	−0.005	−0.010	0.000	0.069
IGF-I								
Intercept	0.714	−0.305	1.734	0.170	−0.728	−0.964	2.419	0.399
Linear change	−0.403	−0.956	0.150	0.154	−0.353	−1.308	0.603	0.469
Quadratic change	0.003	−0.001	0.007	0.163	0.002	−0.004	0.009	0.494

Latent growth curve models estimating intercept level, linear change, and quadratic change in BMI and WHtR in relation to IGFBP-1 and IGF-I level at baseline. Statistically significant estimates in bold. IGFBP-1, insulin and IGF-I were ln-transformed for analyses. Intercept level reflects age 73 years. Age was used as the timescale and all models were adjusted for sex and birth cohort. For IGFBP-1, a second model additionally adjusted for insulin, and a third for familial confounding by comparing associations within and between twin pairs.

*BMI* weight(kg)/height(m)^2^, *WHtR* waist/height, *IGF* insulin-like growth factor, *IGFBP-1* IGF-binding protein-1.

**Fig. 3:**
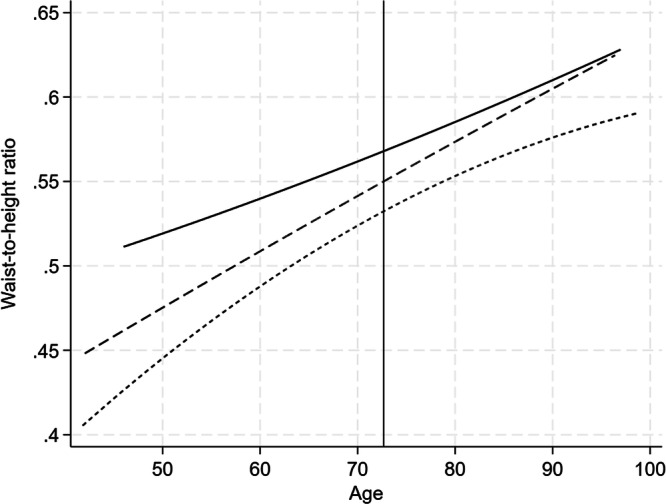
Longitudinal change in waist-to-height ratio, stratified by tertiles of fasting IGFBP-1 concentration. The lowest (ln-IGFBP: 11.946–3.401), middle (ln-IGFBP: 3.434–3.784) and highest (ln-IGFBP: 3.807–5.142) tertile are represented by the solid, broken and dotted line, respectively.

On average, each twin had 4.3 measures of WHtR (range 1–9). WHtR increased from mid- to late life and, in contrast to BMI, continued to increase beyond 70–75 years (Fig. 3). A quadratic model was the best fit. There was a significant inverse relationship between ln-IGFBP-1 and WHtR at the intercept age (Table 3). Every unit increase in ln-IGFBP-1 (equivalent 2.72 μg/L) was associated with a 3.0 cm/m lower WHtR at age 73 years. Adjustment for insulin reduced, but did not abolish, the association. There was no association between ln-IGFBP-1 and the slope or shape of the WHtR trajectory.

In between-within models, the association between ln-IGFBP-1 and intercept level of both BMI and WHtR was lower within twin pairs was smaller than between twin pairs, indicating that part of the association is explained by familial factors (Table 3). However, the associations remained statistically significant also within twin pairs.

There was no association between baseline ln-IGF-I concentration and BMI or WHtR at the intercept age or the slope or shape of the trajectories (Table 3). Therefore, no further analyses of ln-IGF-I were performed.

### Survival

The mean age of death differed between cohorts (Table 1) and there was a trend to poorer survival in twins with lower IGFBP-1 in the 1925–1944 cohort (Table 2). In Cox regression models, individuals were followed on average 19.65 years. No significant differences in survival were observed in relation to baseline ln-IGFBP-1 (hazard ratio 1.00, 95% CI 0.83–1.21) or ln-IGF-I (hazard ratio 1.06, 95% CI 0.78–1.42).

## Discussion

In middle-aged to old-aged twins from the Swedish Adoption/Twin Study of Aging, IGFBP-1 concentrations at baseline were inversely related to levels of adiposity at age 73 but were not predictive of a change in adiposity across mid- and late-life. The relationship was weakened, but not abolished, by adding adjustment for insulin. Between-within regression modeling indicated a greater effect between twin pairs, suggesting that part of the association is explained by familial factors. IGF-I was not associated with levels or trajectories of BMI or WHtR. Neither IGFBP-1 nor IGF-I at baseline predicted survival.

### Baseline characteristics

In our study, insulin, age, and sex explained 27% of the variance in IGFBP-1 concentrations. Unexpectedly, given the well-documented inverse relationship between insulin and IGFBP-1 [15–18], insulin and IGFBP-1 concentrations were both highest in the 1900–1917 cohort i.e., there was an upward shift of the regression line. The higher insulin concentrations in the 1900–1917 cohort, compared to the younger cohorts, is consistent with the insulin resistance of aging [19]. Extreme old age (>90 years) is associated with insulin sensitivity [20]. However, in a study of 72–92-year-old healthy individuals, fasting IGFBP-1 levels were higher, and there was decreased suppression of IGFBP-1 by insulin, compared to those aged 24–58 years old [21]. In another study of men aged 75 or older, IGFBP-1 concentrations were 61% higher than those in men aged 50–54 years [22]. An upward shift in the relationship between IGFBP-1 and insulin has previously been reported in older men (63–81 years compared to 20–39 years) [23], in GH deficiency and type 1 diabetes [24], and in type 2 diabetes [15, 16]. The cause of these shifts has been attributed to relative protein malnutrition and the impact of stimulators of IGFBP-1, including proinflammatory cytokines, as well as hepatic insulin resistance.

The finding that circulating IGFBP-1 is higher in females is consistent with previous research in Swedish individuals aged 35–56 years [16]. In that study, IGF-I concentrations were higher in males, as was observed in our study. Insulin contributed to the variance in IGF-I concentrations, consistent with its direct and indirect effects on IGF-I synthesis in liver [25]. Moreover, we found that IGF-I was lower in older twins, consistent with previous reports of the progressive decline in total IGF-I concentrations in the circulation from young adulthood to old age [26, 27]. At baseline, the oldest twins were 87 years. In healthy centenarians, relatively higher IGF-I concentrations are associated with an better lipid profile than those aged 75–99 years, and may indicate improved insulin sensitivity [28]. There is also a relative excess of IGFBPs in relation to IGF-I with aging [29, 30], which is accompanied by a decrease in IGF-I that is biologically “free” to act at the cellular level [31, 32].

In our study, there was a negative correlation between IGFBP-1 and BMI or WHtR at baseline. Portal insulin concentrations may explain the inverse association between IGFBP-1 and BMI [17, 18], and between other measures of adiposity, including waist measurement [15, 16], skinfold thickness [33], bioelectrical impedance analysis [17] and DEXA [34].

### Trajectories of BMI and WHtR

The age trajectories of BMI and WHtR were both curvilinear. Lower baseline IGFBP-1 concentration was associated with higher BMI and WHtR at the intercept age (set at 73 years), but there was no association between IGFBP-1 and the slope or shape of the BMI or WHtR trajectory. BMI increased in this population up until the age of 70–75 years and then declined, however this was not seen for WHtR, which continued to increase with age. Another study has concluded that associations between BMI and IGFBP-1 is driven by weight gain in early-middle adulthood [35]. Our findings suggest that IGFBP-1 in middle-older age reflects relative adiposity that is maintained across the later part of the lifespan.

### Survival

Previous studies have shown that higher IGFBP-1 is associated with higher all-cause mortality. In a study in a Swedish population of individuals aged 66–81 years followed for 12 years, those in the highest tertile for IGFBP-1 had higher all-cause mortality [36]. In an Italian population over 65 years followed for 8 years, those in the highest quartile had higher risk of death, while the second and third quartiles negatively predicted mortality [37]. In a US population aged over 70, higher IGFBP-1 was associated with greater mortality during a mean follow-up of 6 years [38]. On the other hand, in a study of men aged 70–89 years and a follow-up of 8 years, no association was IGFBP-1 levels with survival was observed [39]. In our study, there was no relationship between IGFBP-1 and survival. Our observation that IGF-I concentrations did not predict survival is consistent with previously published research [38], while others have observed low IGF-I is a predictor of mortality [37].

### Strengths and limitations

The strength of this study lies in the long-term follow-up (more than 30 years), with multiple in-person testing of twin pairs, objective adiposity measures, and mortality information from linkage to registers with nationwide coverage. Twins contributing to this dataset were born between 1900 and 1944, and population trends, including mortality, changed significantly during their lifetimes. Therefore, birth cohort was treated as a covariate in the analyses. Since insulin is an important regulator of IGFBP-1, measurement of both insulin and IGFBP-1 added to interpretation, e.g., of higher levels in the oldest cohort. The inclusion of twins also allowed us to test the associations within twin pairs, to adjust for genetic and other familial confounding. However, there were also limitations. Details of cardiometabolic conditions, e.g., diabetes, which may have influenced the associations between IGFBP-1 and trajectories of BMI or WHtR, were not available for inclusion in the models. Furthermore, we did not measure the concentrations of other regulators of IGFBP-1, e.g., proinflammatory cytokines, that may have contributed independently to the variance in IGFBP-1, only 26% of which was explained in this study. When studied in 1986–1988, the older twin-cohort born 1900–1917 had a mean age of 75 years, and for individuals in Sweden born around 1910, average life expectancy was less than 70 years [8]. Thus, in our study, there was a bias to longevity. IGFBP-I, IGF-I and insulin were measured at just one time point, at baseline. However, these measurements will change with age, adiposity and intercurrent illness.

## Conclusion

In conclusion, lower circulating IGFBP-1, independent of insulin concentrations, are associated with increased adiposity but not change in adiposity, across the lifespan from middle to old age. Factors other than insulin contribute to most of the variance IGFBP-1 concentrations. We speculate that these, e.g., proinflammatory cytokines may contribute to the association between IGFBP-1 and adiposity. The effect within twin pairs was smaller than between twin pairs, indicating that part of the association is explained by familial factors.

## Data Availability

The data on SATSA are archived in the National Archive of Computerized Data on Aging (NACDA) [40].
